# Supplementary material for: Disulfide-activated protein kinase G Iα regulates cardiac diastolic relaxation and fine-tunes the Frank–Starling response
Source: Nat Commun. 2016 Oct 26;7:13187. doi: 10.1038/ncomms13187 (PMC5095173; doi:10.1038/ncomms13187)
Supplement: Supplementary Information — Supplementary Figures 1-2 and Supplementary Tables 1-2. [file ncomms13187-s1.pdf]

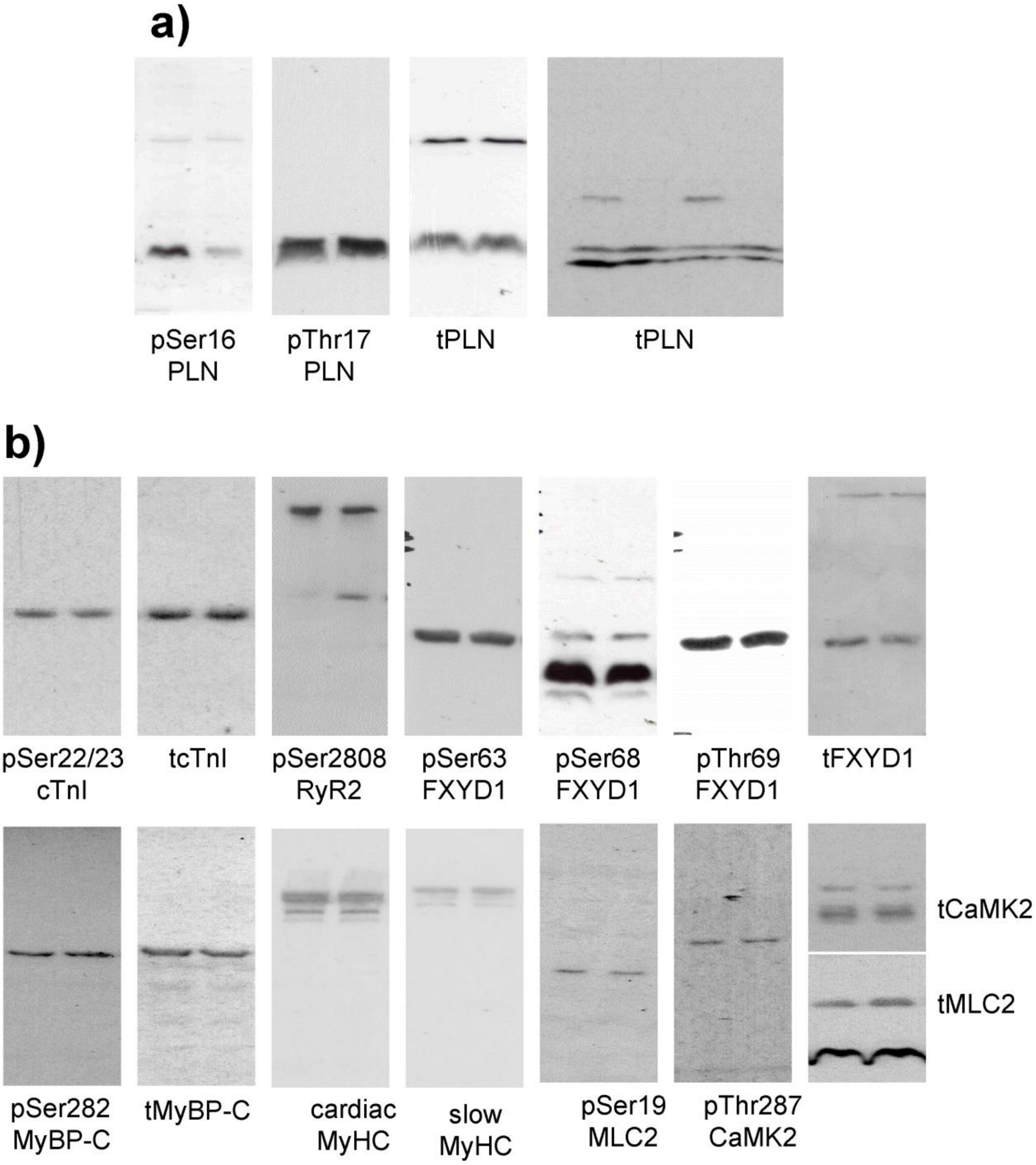

Supplementary Figure 1 Uncropped scanned images of key western blots from Figure 1. Uncropped blots used in Figure 1 (a-b).

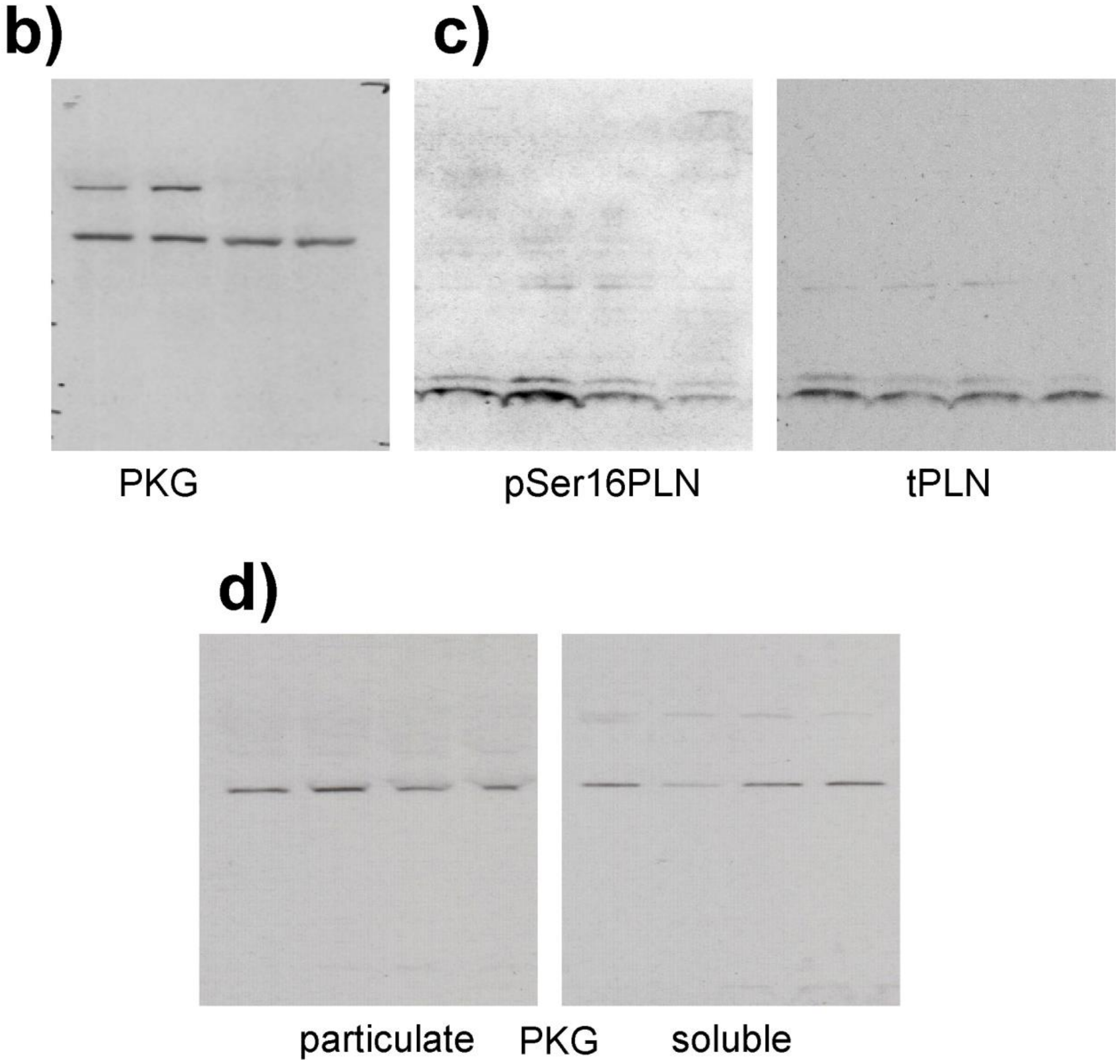

Supplementary Figure 2 Uncropped scanned images of key western blots from Figure 2. Uncropped blots used in Figure 2 (b-d).

Supplementary Table 1. Echocardiography measurements from WT and C42S PKGIα KI mice.

	**WT**	**C42S PKG KI**	
	**n=13**	**n=14**	
Body weight, g	28.2 ± 0.45	27.6 ± 0.30	ns
Heart Rate, bpm	549 ± 5.82	540 ± 4.72	ns
Intraventricular septum diastolic thickness, mm	0.86 ± 0.03	0.82 ± 0.03	ns
LV diastolic dimension, mm	4.22 ± 0.07	3.86 ± 0.07	p<0.01
Posterior wall diastolic thickness, mm	0.86 ± 0.04	0.89 ± 0.06	ns
Anterior wall diastolic thickness, mm	0.86 ± 0.04	0.81 ± 0.03	ns
Intraventricular septum systolic thickness, mm	1.36 ± 0.05	1.25 ± 0.04	ns
LV systolic dimension, mm	2.61 ± 0.08	2.53 ± 0.06	ns
Posterior wall systolic thickness, mm	1.31 ± 0.06	1.31 ± 0.05	ns
Anterior wall systolic thickness, mm	1.32 ± 0.04	1.24 ± 0.06	ns
Diastolic endocardial area, mm^2	25.4 ± 0.91	20.8 ± 0.85	p<0.01
Systolic endocardial area, mm^2	15.3 ± 0.92	13.9 ± 0.3	ns
Diastolic epicardial area, mm^2	42.6 ± 1.14	37.7 ± 0.92	p<0.01
Systolic epicardial area, mm^2	34.1 ± 1.00	32.2 ± 0.48	ns
LV systolic volume, µL	25.5 ± 1.94	23.6 ± 1.54	ns
LV diastolic volume, µL	80.1 ± 3.00	67.1 ± 1.85	p<0.01
Stroke volume, µL	54.5 ± 1.47	43.5 ± 1.17	p<0.0001
Ejection Fraction, %	68.6 ± 1.23	66.52 ± 0.98	ns
Fractional Shortening, %	37.6 ± 1.32	36.7 ± 1.27	ns
Cardiac output, mL/min	29.3 ± 0.79	23.2 ± 0.64	p<0.0001
LV Mass, mg	115.3 ± 2.14	114.5 ± 3.35	ns
AV Peak velocity, mm/s	1876.4 ± 56.2	1798.3 ± 131.8	ns

Ao Velocity time integral, cm	6.06 ± 0.17	5.97 ± 0.50	ns
Transmitral E wave velocity (mm/s)	953.4 ± 19.7	820.9 ± 18.9	p<0.01
Transmitral A wave velocity (mm/s)	519.5 ± 19.4	609.9 ± 19.9	p<0.05
E/A ratio	1.84 ± 0.05	1.35 ± 0.03	p<0.001
Mitral annulus motion e’ wave (mm/s)	31.8 ± 0.58	27.7 ± 0.80	p<0.05
Mitral annulus motion a’ wave (mm/s)	16.9 ± 0.54	18.4 ± 0.42	p<0.05
e’/a’ ratio	1.89 ± 0.09	1.51 ± 0.06	p<0.05

Supplementary Table 2. Pressure-volume measurements from WT and C42S PKGIα KI mice.

	**WT**	**C42S PKG KI**	
	**n=9**	**n=10**	
Body weight, g	28.1 ± 0.8	28.8 ± 1.0	ns
Heart Rate, bpm	551 ± 12.7	563 ± 21.6	ns
End diastolic pressure, mmHg	3.2 ± 0.1	6.0 ± 1.1	p<0.05
End systolic pressure, mmHg	90.5 ± 3.6	91.5 ± 2.9	ns
LV developed pressure, mmHg	101.1 ± 2.5	96.0 ± 2.5	ns
Mean pressure, mmHg	39.0 ± 1.4	46.4 ± 1.5	p<0.05
End systolic volume, µL	13.6 ± 1.5	14.1 ± 2.6	ns
End diastolic volume, µL	34.5 ± 2.3	33.9 ± 3.1	ns
Stroke work, mmHg.µL	2383.8 ± 121.0	2268.5 ± 138.7	ns
Ejection Fraction, %	84.9 ± 4.4	76.3 ± 7.7	ns
dP/dt max, mmHg/s	9603.8 ± 299.6	8528.9 ± 279.5	p<0.05
dP/dt min, mmHg/s	-10624.9 ± 389.4	-8900.6 ± 586.9	p<0.05
Tau (Weiss), ms	5.35 ± 0.23	6.30 ± 0.33	p<0.05
